# Children With Airway Compressions Caused by Mediastinal Tumors: A Single-Center Case Series

**DOI:** 10.7759/cureus.67710

**Published:** 2024-08-25

**Authors:** Ryosuke Nakai, Kazunori Aoki, Taisuke Matsumoto, Norihisa Miyashita, Hiroshi Kurosawa

**Affiliations:** 1 Pediatric Critical Care Medicine, Hyogo Prefectural Kobe Children’s Hospital, Kobe, JPN; 2 Emergency Medicine, Hyogo Prefectural Kobe Children’s Hospital, Kobe, JPN

**Keywords:** sudden death, bronchial asthma, airway obstruction, mediastinal tumor, misdiagnosis

## Abstract

Background: Mediastinal tumors in children may be misdiagnosed as bronchial asthma, resulting in delayed diagnosis and airway obstruction leading to cardiac arrest.

Purpose: This study aimed to identify factors for early diagnosis of mediastinal tumors in children to avoid a life-threatening situation.

Methods: We retrospectively reviewed the medical records of 14 children with airway compressions caused by mediastinal tumors who visited the Hyogo Prefectural Kobe Children’s Hospital between April 2016 and September 2023.

Results: The median age at diagnosis was 9.8 years; all patients had anterior mediastinal tumors. Respiratory symptoms included cough in seven cases, orthopnea in five cases, wheezing in four cases, tachypnea in one case, and no symptoms in one case. Although six patients (42%) visited medical institutions presenting with respiratory symptoms, more than a month passed before diagnosis. Six patients (42%) received bronchial asthma treatment. Most cases (n=9, 64%) were diagnosed using chest radiography; two cases were diagnosed using computed tomography. Three patients experienced cardiac arrest because of airway obstruction before mediastinal tumor diagnosis. Chest radiography on admission showed abnormal mediastinal shadows in all cases.

Conclusion: In children with prolonged respiratory symptoms and an atypical course of bronchial asthma, mediastinal tumors should be considered as a differential diagnosis, and chest radiography should be performed.

## Introduction

Recent studies have shown that 27% of pediatric mediastinal tumors are asymptomatic and discovered incidentally [1]; approximately 60% of patients with clinical symptoms have respiratory symptoms [2]. Respiratory symptoms due to airway compression by mediastinal tumors have been misdiagnosed as bronchial asthma, delaying diagnosis [3,4]. There are reports describing cardiac arrest due to airway obstruction occurring before diagnosis [5,6]. However, little is known regarding the course of diagnosis in life-threatening cases. This report aimed to clarify the clinical course and findings in children with airway compressions caused by mediastinal tumors and to investigate factors for early diagnosis.

## Materials and methods

We retrospectively reviewed the medical reports of children who visited Hyogo Prefectural Kobe Children’s Hospital, Kobe, Japan for mediastinal tumors with airway compressions between April 2016 and April 2023. This study was approved by the Ethical Review Board in Hyogo Prefectural Kobe Children’s Hospital (R5-108).

This study included all patients aged < 18 years at the time of diagnosis. Based on a previous report, airway compression was defined as dyspnea with wheezing and significant airway narrowing of >50% on radiological imaging [7]. The demographic data included age at diagnosis, sex, medical history, anatomical location, diagnosis of the mediastinal tumor, respiratory symptoms, duration from symptom onset to diagnosis, and treatment for respiratory symptoms, as well as treatment in the hospital. The final diagnosis of the mass was based on histological analysis. The outcomes were the incidence of cardiac arrests before diagnosis and survival discharge from the pediatric ICU (PICU).

## Results

The study included 14 children (nine males and five females) with a median age of 9.8 years at diagnosis. One patient (Patient 7) had a medical history of cardiac surgery for an interrupted aortic arch, while the other patient had no particular medical history. Biopsies revealed six cases of lymphoma, three cases of leukemia, two cases of teratoma, one case of neuroblastoma, one case of yolk sac tumor, and one unknown case. The tumor was located in the anterior mediastinum in all the cases (Table 1).

**Table 1:** Patient characteristics, treatments, and outcomes PICU, pediatric intensive care unit; CT, computed tomography; PMLBL, primary mediastinal large B-cell lymphoma; ALCL, anaplastic large cell lymphoma; T-LBL, T lymphoblastic lymphoma; T-ALL, T acute lymphoblastic leukemia; NPPV, non-invasive positive pressure ventilation; N/A, not applicable.

Patient	Age (years)	Sex	Diagnosis	Location of mediastinal tumor	Respiratory symptoms	Other symptoms	Length of time to diagnosis	Test for diagnosis	Diagnosis of bronchial asthma	Bronchial asthma treatment	Oral corticosteroid before diagnosis	Treatment in PICU	Cardiac arrest before diagnosis	Outcome
1	2	F	Teratoma	Anterior	Cough, Wheezing		3 months	-	+	STEP 1	-	Ventilation	+	Death
2	3	F	Neuroblastoma	Anterior	Cough	Cervical lymphadenopathy	1 week	Chest X-ray	-		-	Ventilation	-	Alive
3	6	M	T-ALL	Anterior	Wheezing		1 month	-	+	STEP 2	＋	Ventilation	+	Death
4	7	M	T-LBL	Anterior	Wheezing, Orthopnea		1 month	Chest X-ray	-		-	Oxygen therapy Corticosteroid	-	Alive
5	8	M	ALCL	Anterior	Orthopnea	Fever, Leg pain	3 weeks	CT, Bronchoscopy	-		-		-	Alive
6	8	M	T-ALL	Anterior	Wheezing, Orthopnea		2 weeks	Chest X-ray	+	STEP 2	-	NPPV	-	Alive
7	8	F	T-LBL	Anterior	Cough	Facial edema	2 weeks	Chest X-ray	+	STEP 1	-	Corticosteroid	-	Alive
8	10	M	T-ALL	Anterior	Cough, Orthopnea	Cervical lymphadenopathy	1 month	Chest X-ray	+	STEP 1	-	Oxygen therapy	-	Alive
9	11	M	PMLBL	Anterior	Cough, Orthopnea		2 days	Chest X-ray	-		-		-	Alive
10	14	F	T-LBL	Anterior	Cough		4 months	Chest X-ray	+	STEP 3	＋		-	Alive
11	14	F	Teratoma	Anterior	Tachypnea	Chest pain	2 days	CT	-		-	Oxygen therapy	-	Alive
12	14	M	N/A	Anterior	Cough		2 days	-	-		-	Ventilation	+	Death
13	15	M	Hodgkin lymphoma	Anterior	None			Chest X-ray	-		-		-	Alive
14	18	M	yolk sac tumor	Anterior	Cough	Facial edema	1 month	Chest X-ray	-		-	NPPV	-	Alive

Symptoms and tests leading to diagnosis

The onset patterns included respiratory symptoms (n=11 cases), fever and leg pain (n=1), chest pain (n=1), and asymptomatic (n=1). Respiratory symptoms included cough (n=7), orthopnea (n=5), wheezing (n=4), and tachypnea (n=1).

Although six patients (42%) visited medical institutions presenting with respiratory symptoms, more than one month had passed since diagnosis; further, six (42%) cases were treated for bronchial asthma. Step one of the Global Strategy for Asthma Management and Prevention [8] was performed in three cases, Step two in two cases, and Step three in one case. Two patients received oral corticosteroids for bronchial asthma exacerbation. Patients without a diagnosis of bronchial asthma presented with symptoms such as leg pain, chest pain, facial edema, and cervical lymphadenopathy, in addition to respiratory symptoms, which led to the diagnosis of mediastinal tumors.

Chest radiography was the most common test for recognizing mediastinal tumors in nine cases. Patient 5 presented with fever, left thigh pain, and orthopnea. A biopsy was performed under general anesthesia; however, ventilation failure occurred after intubation. Bronchoscopy and CT revealed airway compression caused by a mediastinal tumor. Patient 11 had intermittent chest pain but started experiencing tachypnea with hypoxemia; consequently, a tumor was detected on computed tomography.

Cardiac arrest cases

Three patients (Patients 1, 3, and 12) experienced sudden cardiac arrest because of airway obstruction before diagnosis; all these patients died. Patient 1 experienced shortness of breath on exertion for three months and had been diagnosed with bronchial asthma by a family doctor. The cough got worse two days before cardiac arrest at home. After 40 minutes of cardiopulmonary resuscitation, the patient returned to spontaneous circulation. Semi-emergency tumor resection was performed; however, the patient died of hypoxic-ischemic encephalopathy on day 45 of hospitalization. Patient 3 experienced wheezing for one month. He was treated with leukotriene receptor antagonists and oral corticosteroids for bronchial asthma by a primary physician; however, his respiratory symptoms showed little improvement. His breathing difficulties gradually worsened, and he had a cardiac arrest at home. Spontaneous circulation returned after 40 minutes of cardiopulmonary resuscitation, but he died the next day. Patient 12 had a cough for two days and experienced cardiac arrest after going to bed at home. The patient died without responding to cardiopulmonary resuscitation. Chest radiography after admission revealed abnormal mediastinal shadows in all patients.

Hospital treatment and outcome

Three patients received oxygen therapy, and two required noninvasive positive pressure ventilation (NPPV). Four patients were on mechanical ventilation, including three patients who had cardiac arrest. Five patients did not require any respiratory support. In two patients, corticosteroids were administered for emergency oncology. Although 11 patients survived to be discharged from the PICU, three patients suffered cardiac arrest before the mediastinal tumor was diagnosed; all of whom died.

## Discussion

Misdiagnosis of bronchial asthma

In this study, six patients (42%) were diagnosed with bronchial asthma before a mediastinal tumor was recognized, receiving bronchial asthma treatment, including oral corticosteroids. Three patients experienced cardiac arrest and died. None of the patients diagnosed with bronchial asthma experienced a recurrence of bronchial asthma exacerbation after therapy for mediastinal tumors. Therefore, the respiratory symptoms recognized as bronchial asthma were assumed to be caused by the mediastinal tumor compressing the airway. In our cases, some were diagnosed with mediastinal tumors from diminished breath sounds in one lung, but most cases presented stridor, wheeze, or no abnormalities on auscultation, making it difficult to diagnose mediastinal tumors based on auscultation findings. 

 The median age at diagnosis of mediastinal tumors in this study was 9.8 years, similar to previous reports (10-13 years) [9-11]. In contrast, the age of bronchial asthma onset in children is two to three years, recently reported to be decreasing [12]. When patients with first-onset bronchial asthma are older than the common age, mediastinal tumors should be considered in the differential diagnosis. 

Risk of administrating corticosteroids without recognizing mediastinal tumors

Two patients (Patients 3 and 10) who were administered oral corticosteroids to treat bronchial asthma exacerbation took long periods (four months, one month) to get a diagnosis; Patient 3 had cardiac arrest before being diagnosed. Their tumors were T-lymphoblastic lymphoma and T-acute lymphoblastic leukemia; corticosteroids temporarily reduced the tumor size, possibly improving the respiratory symptoms and delaying diagnosis. In our cases, corticosteroid administration did not affect the biopsy results. Corticosteroid administration before biopsy results in an inaccurate histological diagnosis or delayed definitive diagnosis in 22% of cases [13]. Moreover, a case of tumor lysis syndrome reportedly occurred after only a single dose of corticosteroids [14]. Administering corticosteroids for bronchial asthma may cause unexpected adverse events; therefore, including mediastinal tumors in the differential diagnosis is important when administering long-term corticosteroids.

The usefulness of chest X-rays

The detection rate of mediastinal tumors by chest X-rays is 97% in adults [15]. In a study of 29 children with mediastinal tumors, abnormal chest radiography findings were reported in all cases [16]. Abnormal shadows in the mediastinum were observed in all patients in our study; early diagnosis was possible using chest radiography. Chest radiography should be considered in cases with poor response to standard treatment for bronchial asthma, abnormal breathing patterns such as orthopnea, and the long-term administration of corticosteroids.

Cases requiring cardiopulmonary resuscitation

All patients who suffered cardiac arrest died; in all cases, tumors were located in the anterior mediastinum. Airway obstruction was not relieved during cardiopulmonary resuscitation because of the tumor’s weight in the supine position. Therefore, children with airway obstruction compressed by mediastinal tumors are less likely to return to spontaneous circulation; providing sufficient ventilation may be difficult even if tracheal intubation is successful. Patients should be transported to a facility proficient in airway management, including extracorporeal membrane oxygenation. Diagnosis before cardiac arrest is the most important step for survival.

## Conclusions

Mediastinal tumors may be misdiagnosed as bronchial asthma, resulting in delayed diagnosis and airway obstruction leading to cardiac arrest. Chest radiography on admission showed abnormal mediastinal shadows in all cases. In children with prolonged respiratory symptoms and an atypical course of bronchial asthma, considering mediastinal tumors as differential diagnoses and performing chest radiography are important.
